# Editor's Note

**DOI:** 10.1179/107902611X13046044199084

**Published:** 2011-05

**Authors:** Donald Bodner

This issue marks *The Journal of Spinal Cord Medicine's* third with our new international publisher, Maney Publishing. I am pleased to report that the feedback on *JSCM*'s new look has been overwhelmingly positive. Of greater importance is that with Maney's expanded online article access, the journal is more widely available to professionals and libraries around the world.

For the journal and the dynamic field of spinal cord injury, there's never been a more exciting time. The broad range of research and clinical activities of interest to our readers is evident in the 2011 meeting agendas for the two organizations that feature *JSCM* as a benefit for their members—the Academy of Spinal Cord Injury Professionals (the Academy) and the American Spinal Injury Association (ASIA).

From June 4 to 8, ASIA joins with the International Spinal Cord Society (ISCoS) for their shared meeting in Washington, DC—*ISCoS-ASIA 2011 International Conference on Spinal Cord Medicine and Rehabilitation*. More than 750 scientists and clinicians will be in attendance for the joint conference program, as well as the *Spine Symposium* on June 4 and the pre-course, *The State of the Science in Spinal Cord Injury Rehabilitation*, beginning June 5.

Las Vegas, Nevada is once again the site for the Academy's annual conference, this time hosted at Rio All Suites Resorts and Casino from September 5-7. Look for the return appearance of Dr. Vernon Lin in *SCI Medicine - An Intensive Review Preconference Workshop*. This comprehensive review of assessment and treatment and rehabilitation strategies is highly recommended, especially for those preparing for the Subspecialty SCI Certification and Maintenance of Certification Examination offered by the American Board of Physical Medicine & Rehabilitation.

Make time in your schedule for these terrific opportunities for education, inspiration, networking and relaxation. *JSCM* looks forward to bringing you interdisciplinary content reflecting the diverse topics addressed in Washington, DC, and Las Vegas.

Comments and suggestions from our readers are always welcome.

